# Effect of a Constant Magnetic Field on Cell Morphology and Migration Mediated by Cytoskeleton-Bound Magnetic Nanoparticles

**DOI:** 10.3390/ijms26115330

**Published:** 2025-06-01

**Authors:** Olga Karavashkova, Artem Minin, Alexandra Maltseva, Pavel Tin, Georgy Nosov, Alexander M. Demin, Nelly S. Chmelyuk, Maxim Abakumov, Valeria Tsvelaya, Victoria Shipunova, Anastasiia Latypova, Ilya Zubarev

**Affiliations:** 1 Moscow Center for Advanced Studies, Kulakova Str. 20, Moscow 123592, Russia; okaravashkova@gmail.com (O.K.); yersiniapestis@inbox.ru (A.M.); pavel_tin@mail.ru (P.T.); vts93@yandex.ru (V.T.); vika_shipunova@mail.ru (V.S.); ana.a.latypova@gmail.com (A.L.); 2 M.N. Mikheev Institute of Metal Physics, Russian Academy of Sciences (Ural Branch), Kovalevskaya Str, 18, Ekaterinburg 620108, Russia; 3 Federal Center of Brain Research and Neurotechnologies, Federal Medical Biological Agency, Ostrovitianov Str, 1, Moscow 117997, Russia; nosov.g@fccps.ru; 4 Life Improvement by Future Technologies (LIFT) Center, Skolkovo, 5, Nobel Str., Moscow 121205, Russia; 5 Postovsky Institute of Organic Synthesis, Russian Academy of Sciences (Ural Branch), Ekaterinburg 620990, Russia; amd2002@mail.ru; 6 Department of Medical Nanobiotechnology, Pirogov Russian National Research Medical University, Ostrovitianov Str., 1, Moscow 117997, Russia; nellichmelyuk@yandex.ru; 7 Laboratory of Biomedical Nanomaterials, National Research Technological University “MISIS”, Leninsky Prospekt, 4, Moscow 119049, Russia; abakumov.ma@misis.ru

**Keywords:** magnetogenetics, nanoparticles, cytoskeleton, cell morphology

## Abstract

Cell migration, shape maintenance, and intracellular signaling are closely linked to dynamic changes in cell morphology and the cytoskeleton. These processes involve the reorganization of the cytoskeleton within the cytoplasm, affecting all its key components: intermediate filaments, microtubules, and microfilaments. A promising strategy for remotely controlling cellular functions is the use of magnetic nanoparticles, which can influence cellular physiology. This approach, known as magnetogenetics, has been applied in various areas of cell and molecular biology. Applying a magnetic field allows for the non-invasive modulation of biochemical processes, cell migration, and morphological changes in cells containing magnetic nanoparticles. In our study, magnetic nanoparticles were conjugated with antibodies targeting cytoskeletal components, enabling the magnetically induced manipulation and deformation of the cell cytoskeleton. Our research introduces a novel approach to manipulating specific cytoskeletal components and altering cell polarity with spatial precision in vitro using magnetic nanoparticles associated with the cytoskeleton.

## 1. Introduction

Magnetic nanoparticles (MNPs) are widely utilized in biological research and appear to be promising actuators of biological processes [1,2]. While this research has yet to be translated into clinical practice, MNPs remain valuable for investigating a wide range of biological processes.

The application of a magnetic field combined with magnetic nanoparticles enables the non-contact manipulation of cell motility [2]. Mechanical forces exerted by magnetic nanoparticles induce cellular tension, partially mimicking in vivo conditions [3]. This approach allows for the regulation of cell positioning on plastic, as well as the control of migration speed and direction [4], mediated by traction forces at the cell periphery [5]. Such directed cell migration is particularly significant for guiding the growth of neurons and Schwann cells [6]. Additionally, studies have proposed the use of magnetic nanoparticles to create and spatially manipulate magnetic multicellular spheroids [7].

Using magnetic nanoparticles and electromagnetic tweezers, it is possible in vitro to determine the viscosity of the cytoplasm of a living cell, which is important for modeling the behavior of nanoparticles in cells and determining the physical characteristics of cells [8]. Ferrofluid-based magnetic tweezers can be employed to analyze the rheological properties of the complex of microtubules and actin associated with each other throughout the cytoplasm [9]. The delivery of exogenous magnetic agents into cells often leads to endosomal uptake, thereby limiting the effectiveness of manipulating subcellular structures. The genetically controlled biomineralization of iron oxide cores has been suggested as a method to enable the magnetic manipulation of cells without the delivery of exogenous agents [10].

In studying the physical and biochemical properties of cells, it is crucial to understand how individual cellular components respond to physical stimuli. Magnetic nanoparticles can nonspecifically bind and remodel the cytoskeleton, leading to alterations in the mechanical properties (such as rigidity) of cells [11]. The interaction of nanoparticles with the cytoskeleton can be used to selectively treat cancer cells by disrupting the cytoskeleton [12] or affecting entire cells and cell–cell interactions [13]. Many methods and materials have been proposed to disrupt the cytoskeleton using nanoparticles [14]. For instance, encapsulating magnetic cobalt–platinum nanoparticles within microtubules has enabled the magnetically induced alignment of microtubules outside cells [15]. Despite these advancements, magnetic nanoparticles have not yet been widely applied to address fundamental questions in cytoskeleton biology. In our work, we are the first to propose an approach to the selective manipulation of various components of the cytoskeleton (intermediate filaments, microfilaments, and microtubules) to study cell behavior and clarify the role of individual components in the processes of spreading and migration.

## 2. Results

### 2.1. Affinity of Chemically Cross-Linked Antibodies with Nanoparticles

We have demonstrated the safety of antibody affinity after conjugation with nanoparticles using paper chromatography. Different ratios of antibodies to bovine serum albumin (BSA) and iron nanoparticles pass differently in the chromatographic strip depending on the ratio of the components (Figure S1 in Supplementary Material). The presence of bands indicates the specific binding of the nanoparticle–BSA antibody complex to the BSA protein. The crosslinking of 6.25 µL MNPs with 10 µL antibodies for 30 min was more efficient than crosslinking with 4 µL; after incubation for 1 h, the band intensities became equal (Figure S1 in the Supplementary Material). After half an hour, fewer antibodies were bound than would have been possible with a given antibody concentration (4 µL). Increasing the crosslinking time allowed for more antibodies to bind specifically. No bands were detected on control membranes. Due to the formation of iron nanoparticle aggregates in solution, only a small portion of the antibody-bound nanoparticles move in the mobile phase. This may indicate the low efficiency of direct cross-linking and the instability of nanoparticles. Increasing the amount of antibodies decreases the bending intensity, possibly due to the cross-linking of intermolecular proteins on the surface of the nanoparticles. Subsequently, crosslinking was performed with a number of antibodies (4 µL) for 1 h without loss of efficiency.

We were unable to visualize the cell cytoskeleton using the direct cross-linking of antibodies with nanoparticles and dye. Therefore, an indirect crosslinking method through additional antibody recognition proteins was used. To stain the cytoskeleton in vitro, we proposed cross-linking the nanoparticles with protein G, which can recognize and strongly bind primary antibody chains [16]. However, the use of separate labeling did not allow for the detailed visualization of the cytoskeleton in cells (Figure S2 in the Supplementary Material). Microtubules, actin, and vimentin filaments were labeled using primary and secondary antibodies (Figure S3 in the Supplementary Material).

### 2.2. Lysis Experiment

**Figure 1:**
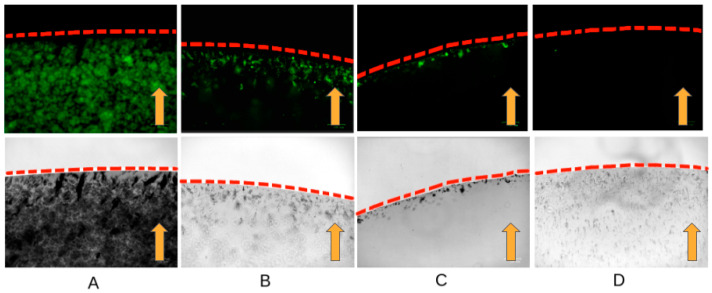
Lysis experiment. The red dotted line shows the border of the drop of the cell lysate. The orange arrows indicate the magnetic field direction. (**A**) Antibodies to alpha-acetyl-tubulin; (**B**) antibodies to vimentin; (**C**) antibodies to actin; (**D**) control without antibodies.

To confirm the safety of the affinity of the cross-linked antibodies with nanoparticles inside living cells, experiments were carried out with lysed cells. In live cells, the viscosity of the cytoplasm, the mechanical properties of the plasmalemma, and the spatial organization can prevent binding to the cytoskeleton and movement toward the magnet. In the cell lysate, specific binding and pulling towards the magnet can be clearly demonstrated. We have shown that antibodies cross-linked with nanoparticles retain their affinity and specifically bind to the cytoskeleton in the cell lysate. In the presence of a magnetic field, the magnetization of the fluorescently labeled antibodies bound to the magnetic nanoparticles is observed at the edge of the droplet near the magnet (Figure 1). Probably due to the greater stability of intermediate filaments with the same number of cells, the abundance of fluorescent aggregates in the experiment with vimentin is greater in contrast to actin and tubulin (Figure 1A).

### 2.3. Vertical Gradient Magnetic Field for the Accelerated Uptake of Nanoparticles by Cells

During active uptake, nanoparticles can accumulate in lysosomes, without specific binding to the cytoskeleton [17]. To avoid the endosomal pathway, magnetofection can be used for “dragging” magnetic particles into cells in a strong magnetic field. We applied a magnetic field with a vertical gradient to “drag” the nanoparticles inside the cells. This process was monitored using nuclear magnetic resonance (NMR) relaxometry. NMR relaxometry with hydrogen protons measures the relaxation time of water, which changes in the presence of ferromagnetic objects. Since cells occupy an extremely small part of the liquid volume, it is not possible to directly measure the amount of nanoparticle uptake. Therefore, measuring the residual concentration in the culture medium makes it possible to reliably determine the amount of nanoparticles in the cells. This method allows for the concentration of stable, non-agglomerating nanoparticles to be measured with high sensitivity.

**Figure 2:**
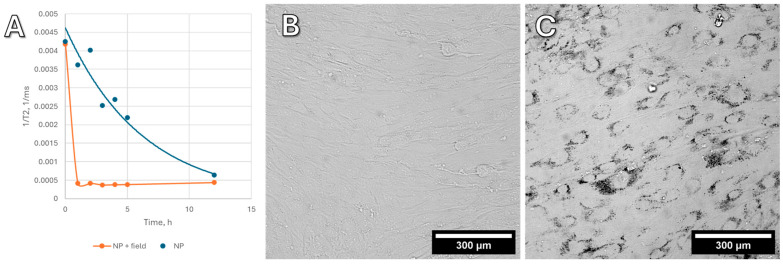
The process of cell uptake of magnetic Fe_3_O_4_ nanoparticles modified with carboxyl groups by ASC52telo culture cells. (**A**) Graphs showing nanoparticle uptake rate with (orange) and without a magnetic system with a vertical gradient. (**B**) Cells before nanoparticle uptake. (**C**) Cells after nanoparticle uptake. Dark areas are nanoparticle aggregates on the cell surface and inside the cells.

Figure 2A shows the change in the relaxation time T2 of hydrogen protons in the culture medium, presented as 1/T2. This value is linearly related to the concentration of nanoparticles in the liquid. Blank experiments were also carried out without cells, and the absorption of nanoparticles onto the plastic of the Petri dish does not make a contribution to this process; all changes in the concentration of nanoparticles are associated with MNP active uptake by the cells. In high concentrations, MNPs (on the order of 0.1–0.5 mg/mL in cells) are visible under a microscope (Figure 2B,C), whereas in small concentrations, only NMR relaxometry allows for monitoring the uptake of nanoparticles.

Without a magnetic field, the uptake of nanoparticles by cells is slow and gradual. In a vertical gradient magnetic field, however, nanoparticles are rapidly absorbed by cells within the first 10–15 min, without any visible change in concentration thereafter. Based on these findings, all subsequent cell experiments included preloading (“dragging”) nanoparticles into the cells using a vertical gradient magnetic field.

In experiments on the uptake of Fe@C (carbon-encapsulated iron nanoparticles) and Fe_3_O_4_ (iron (II, III) oxide) nanoparticles into cells, as well as the lysis experiment, similar results were obtained. However, it was unexpected that Fe@C with similar magnetic characteristics had no visible effect on migration and changes in cell morphology. For this reason, we chose Fe_3_O_4_ nanoparticles as the main ones for this study.

### 2.4. Cell Morphology and Migration Experiments

A series of in vitro experiments were carried out to demonstrate the possibility of changing cell morphology, orientation, and migration speed in the direction of a constant magnetic field gradient (Figure S4 in Supplementary Material). In a magnetic field, the complex ((Fe_3_O_4_)–dye (Cy5)–Protein G–antibody) mechanically affects the cytoskeleton. After pretreating the cells with monodansylcadaverine, the nanoparticles were stabilized and aggregated less along the magnetic field lines. This is important for the specific binding of cytoskeletal fibrils through antibodies. A strong vertical magnetic field “drags” the complex with magnetic nanoparticles into the cells, helping to bypass the endosomal pathway.

**Figure 3:**
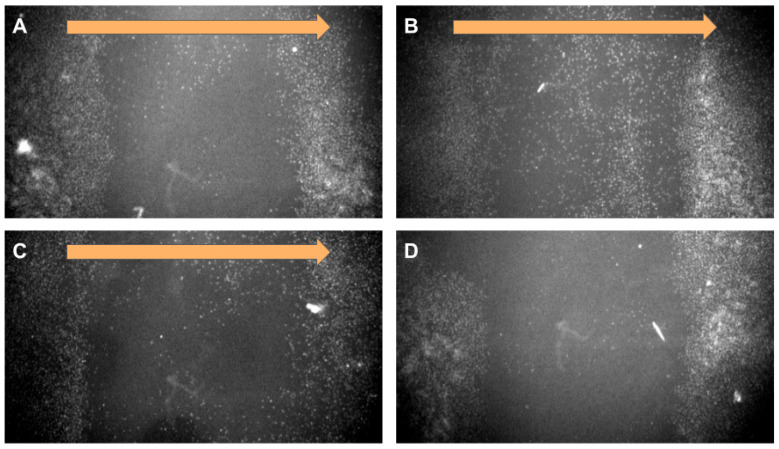
Human bone marrow mesenchymal stem cell (BM MSC) migration in magnetic field. The orange arrow indicates the magnetic field direction. (**A**) BM MSC incubated on a magnetic system, nanoparticle concentration 34,5 µg/mL. (**B**) BM MSC incubated on a magnetic system, nanoparticle concentration 172 µg/mL. (**C**) BM MSC incubated on a magnetic system, nanoparticle concentration 172 µg/mL. The cells were pretreated with integrin antibody solution before being placed in a magnetic system. (**D**) BM MSC incubated without a magnetic system, nanoparticle concentration 172 µg/mL.

In a lateral magnetic field, cells with nanoparticles migrate rapidly toward the magnet (Figure 3). Due to the inability to automatically photograph cells at specific time intervals, we present the results at the starting and ending points. Accelerated migration in a magnetic field may indicate the presence of a pulling force on the cells toward the magnet. Loading cells with a large number of magnetic nanoparticles with or without antibodies does not affect the degree of deformation in a magnetic field but does accelerate the migration rate.

**Figure 4:**
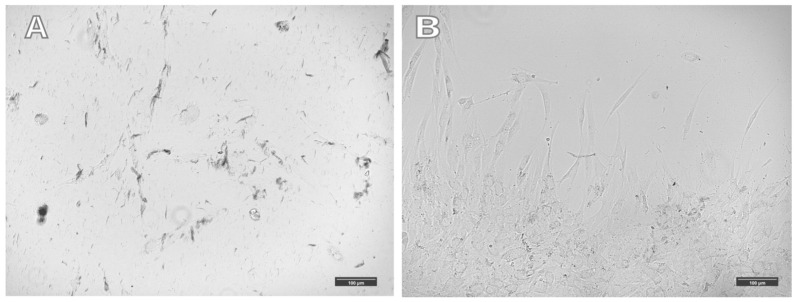
Cells were incubated in a magnetic system with a horizontal magnetic field for 20 h. (**A**) concentration of nanoparticles, 182.16 μg/mL. (**B**) Concentration of nanoparticles, 26.2 μg/mL. The magnetic field is directed upwards.

**Figure 5:**
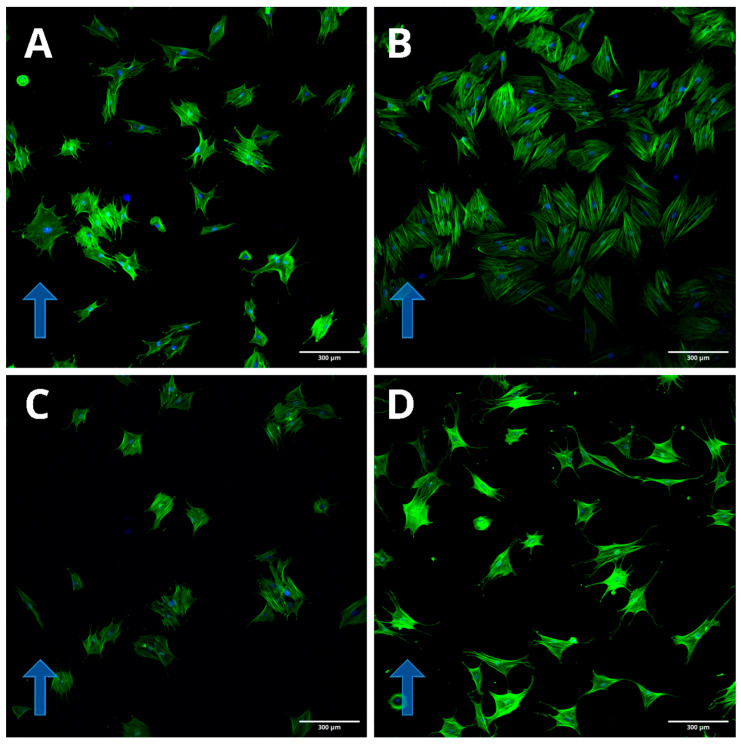
Different morphology of 174H cells. The blue arrow shows the direction of nanoparticle movement in a magnetic field. Confocal microscopy of cells incubated on a magnetic system with a horizontal magnetic field. Cells were incubated with magnetic nanoparticles conjugated with protein G and different antibodies. Labeling was performed with phalloidin. (**A**) Antibodies to vimentin. (**B**) Antibodies to beta-actin. (**C**) Antibodies to tubulin. (**D**) No antibodies; nanoparticles were conjugated with BSA. The magnetic field is directed upwards.

An increase in the number of nanoparticles in cells does not lead to significant changes in cell morphology in a magnetic field. In a magnetic field, cells with magnetic nanoparticles associated with the cytoskeleton antibody migrate rapidly towards the magnet. However, they are not “drawn” toward the magnet and do not change their morphology predominantly. Uploading most of the cytoplasmic mass with magnetic nanoparticles does not lead to significant effects of the magnetic field on cell morphology, and the cells are not “drawn” toward the magnetic field. Large aggregates of magnetic nanoparticles are observed in such cells, aligned along the magnetic field lines. All visible clusters of nanoparticles are aggregates; single nanoparticles cannot be seen under an optical microscope (Figure 4). Cell orientation was clearly visible by staining with labeled phalloidin (Figure 5).

To study the impact of magnetic nanoparticles conjugated with antibodies targeting cytoskeletal components on cell morphology, the modified nanoparticles were delivered into cells using a vertical magnetic system. The cells were then placed into the magnetic system with a lateral field and were incubated for 20 h. The experiment was performed in three variations: (1) cells were placed in a horizontal magnetic field right after nanoparticle uptake; (2) cells were pretreated with alpha-3 integrin antibodies before the nanoparticles were added (the presence of integrin in the cells was confirmed by immunocytochemistry (Figure S5), and after nanoparticle uptake, the cells were placed in a horizontal magnetic field); (3) cells were pretreated with alpha-3 integrin antibodies, and after nanoparticle uptake, the cells were reseeded to a new cultural dish before being placed in a horizontal magnetic field.

The cells used in the experiment have a good ability to migrate. To determine the role of the magnetic field and the pulling effect of the complex of magnetic nanoparticles on cells, we restricted the migratory ability in a series of experiments. It has been suggested that cell contact with the bottom of the culture dish and the extracellular matrix (ECM) limits the cells’ response to the forces acting on nanoparticles from a magnetic field (therefore, acting on cells). We pretreated the cells with an alpha-3 integrin antibody to diminish the role of bounds with ECM. The presence of integrin alpha-3 receptors was determined by immunofluorescence (Figure S5). We assumed that this would reduce the cells’ ability to migrate. Instead of migrating toward the magnet, the cells would be deformed due to the forces from the magnet acting on the nanoparticles captured by cells.

In the third experiment, the cells were reseeded after the magnetic nanoparticles were “dragged” into them. As a result, the formation of contacts with the cultured plastic was reduced. This allowed us to influence the morphology and physiology of cells when contact with the plastic was not very strong.

In each experimental group, we used nanoparticles conjugated with protein G and modified with one of the antibodies to cytoskeleton protein (beta-actin, tubulin, or vimentin). Additionally, we used BSA instead of antibodies in several samples to study if there was any specific role of antibodies or whether the influence on cell morphology is conditioned by magnetic nanoparticles alone. We also used unmodified nanoparticles, conjugated only with protein G to estimate its role. There were samples with nanoparticles conjugated with protein G and modified with antibodies to beta-actin, but the cells were incubated without a magnetic field after nanoparticle uptake. Additionally, we performed the same image analysis on images of cells that were not exposed to any treatment and that were simply fixed and stained in the same way as the experimental samples.

**Figure 6:**
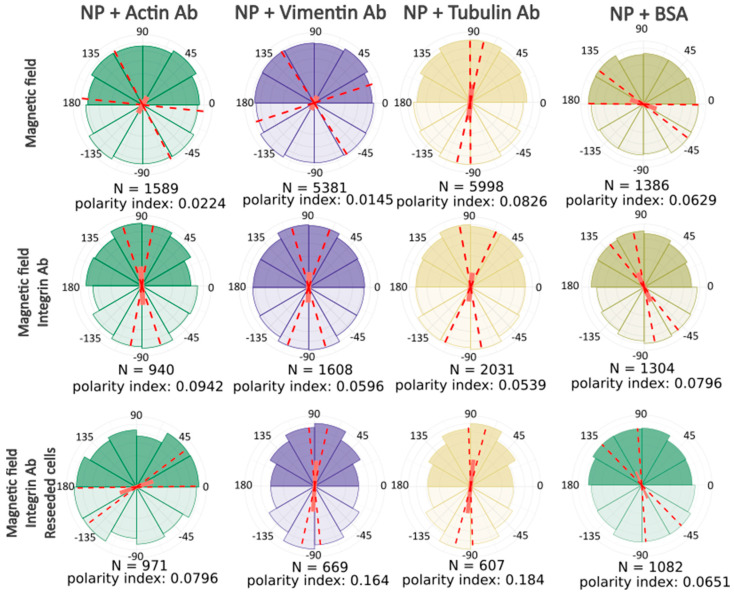
Ensemble plots showing cell polarity for different experimental conditions. 174H cells. Red dashed lines indicate the 95% confidence intervals.

**Figure 7:**
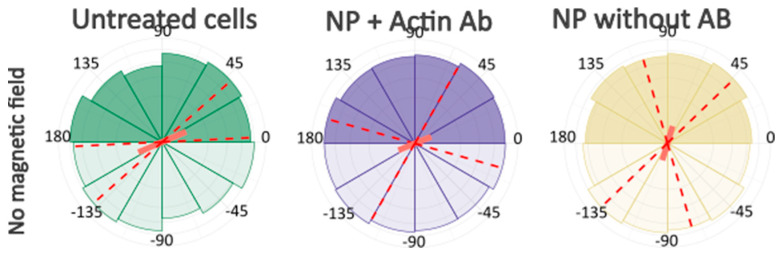
Ensemble plots showing cell polarity for control samples incubated without lateral magnetic field. 174H cells. Red dashed lines indicate the 95% confidence intervals.

**Figure 8:**
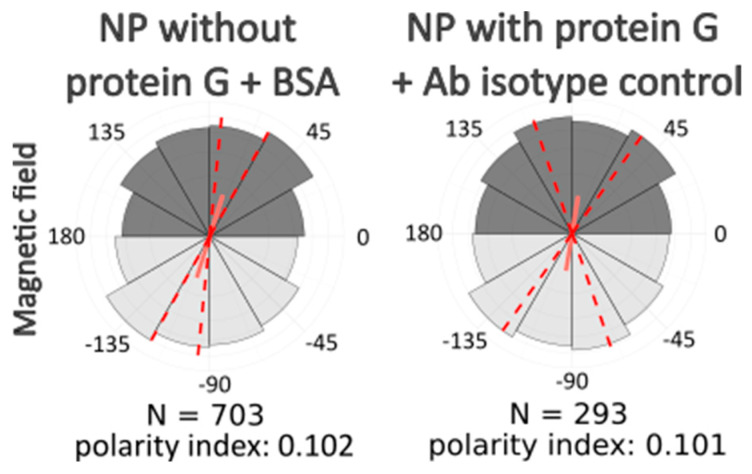
Ensemble plots showing cell polarity for different experimental conditions, control samples incubated with magnetic field and without antibodies to the cytoskeleton components. 174H cells. Red dashed lines indicate the 95% confidence intervals.

After computer processing of the images via Polarity-Jam [18], we obtained data in the form of the orientation of each cell (Figure 6, Figure 7 and Figure 8). The polarity index is the value that is calculated as the length of the mean resulting vector of the directional values of individual cells. This value varies from 0 to 1 and represents how much distribution is concentrated around the mean values. The red dotted lines represent the 95% confidence interval. A polarity index of 1 indicates that all cells are oriented in the same way, while an index of 0 indicates that the data are randomly or evenly distributed.

Without a magnetic field, cells do not have a directional migration or orientation of cells along one of the axes. All cells are randomly oriented and exhibit different shapes and sizes. When exposed to a magnetic field, cells experience mechanical tension from the nanoparticles. Despite this, most experimental samples do not alter the morphology and orientation of the cells. Only in the passage experiments was an effect on the cell orientation revealed. In these cells, the main axis is directed towards the magnetic lines.

This result clearly demonstrates that the basic response of cells to mechanical tension towards a magnet is increased migration rather than a change in cell shape and orientation.

### 2.5. Magnetic Spheroid Experiment

**Figure 9:**
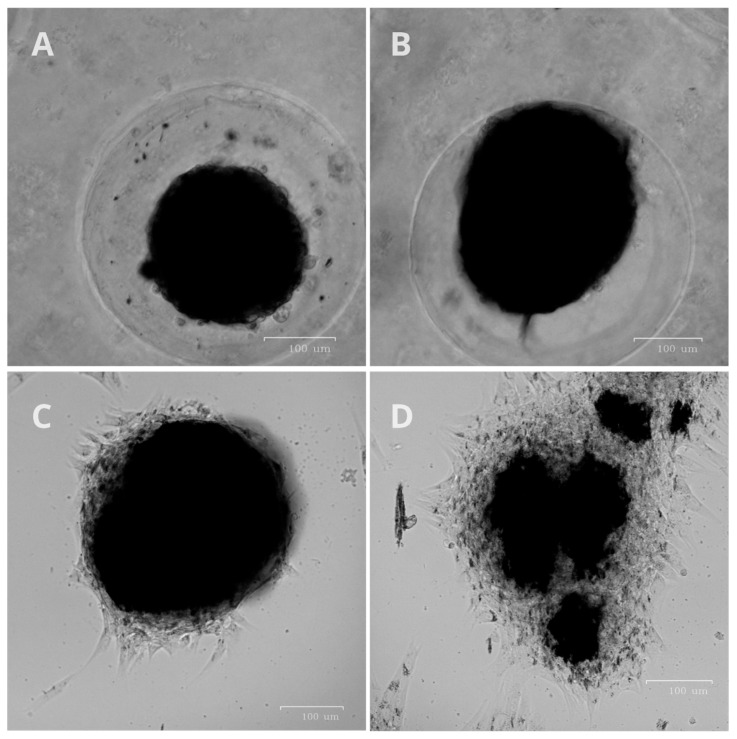
Magnetic spheroid in agarose mold and spreading on the surface of an adhesive Petri dish. (**A**) Magnetic spheroid in an agarose mold without a magnetic field. (**B**) Magnetic spheroid in an agarose mold in a magnetic field. (**C**) Magnetic spheroid on the bottom of a Petri dish without a magnetic field. (**D**) Magnetic spheroid on the bottom of a Petri dish in a magnetic field. Aggregates of magnetic nanoparticles aligned along the magnetic field lines and minor deformation of the magnetic spheroid are visible. Main mass of the spheroid towards the magnet (magnet on top); nanoparticles in the cells are arranged by the magnetic field into large needle-like aggregates.

Spheroids loaded with magnetic nanoparticles were used to assess the possibility of the deformation of large cell masses in three-dimensional cell cultures. In a 3D cell culture, unattached spheroids are magnetized to a magnetic field. In the experiment, the focus was to affect the total mass of the spheroid at the initial stage of attachment to the surface, not after it had completely spread onto the plastic. Once a sufficient number of contacts were formed with the plastic surface, the spheroid stopped stretching toward the magnet. Only the accelerated migration of peripheral cells toward the magnet was then observed. After attachment to plastic, the spheroids were slightly stretched toward the magnet and showed the presence of many aggregates of nanoparticles in the cells, aligned in the direction of the magnet (Figure 9).

## 3. Discussion

When using exogenous magnetic nanoparticles, it is important to ensure that the affinity of antibodies cross-linking to the nanoparticles is retained. A high concentration of primary antibodies during crosslinking results in a decreased band intensity due to intermolecular cross-linking between antibodies on the nanoparticle surface. Optimal protein concentration does not guarantee the preservation of antibodies for the immunohistochemistry of the individual cellular components. The direct cross-linking of nanoparticles, dye, and primary antibodies does not allow for high-quality visualization of the cell cytoskeleton, making such direct cross-linking unsuitable for manipulating cytoskeletal filaments. On the other hand, we have demonstrated that the indirect conjugation of antibodies using a nanoparticle–dye–protein G complex enables efficient binding to antigen epitopes in fixed cells and effective visualization of the cytoskeleton. The efficiency of the indirect cross-linking approach can be explained by the fact that protein G saves activity after chemical modifications and cross-linking.

The affinity of antibodies may differ between a buffer solution and in the cell cytoplasm. An experiment involving cell lysis shows that antibodies retain affinity after being cross-linked with nanoparticles and can magnetize cytoskeletal components from the cell lysate under a magnetic field. This system is not limited by the dense intracellular packing of proteins, which allows for greater flexibility for manipulating cytoskeletal fibrils. It also ensures better recognition and binding between antibodies and antigens while avoiding problems related to antibody degradation by lysosomal enzymes during endocytosis. In the presence of a magnetic field, the cytoskeletal components labeled with antibodies, nanoparticles, and dye, as well as cellular debris, are magnetized toward the magnet. This confirms that in the cytoplasm of lysed cells, antibodies cross-linked with nanoparticles can bind to the cytoskeleton and undergo magnetically controlled movement along a magnetic field gradient.

Cells in a magnetic field are exposed to mechanical forces that pull them towards the magnet due to the presence of magnetic nanoparticles in the cells. These forces can result in two outcomes: pulling cells towards the magnet or accelerating cells’ migration. The increase in cell migration is possible due to the pulling effect on the cells’ leading edges and the general mechanical tension toward the magnet. At the same time, adhesion cell cultures cannot exist in suspension. They require contact with the bottom of the culture vessel. The absence of adhesive contact with the surface is a signal for apoptosis. Contacts with the culture-treated surface of the culture vessel are provided by various integrin protein molecules. Integrins form strong contacts with the substrate and cells [19]. It is important that the maximum possible intracellular amount of magnetic nanoparticles is insufficient for cells to reproducibly elongate in a magnetic field. Using stronger magnets than those presented in our work tears the cells from plastic.

Mechanical tension of the cytoskeletal fibrils deforms the shape and polarity of the cell, slightly extending it in the direction of the magnetic field. Aggregates of magnetic nanoparticles aligned along the magnetic field are clearly visible in the cytoplasm of cells. When changing cell morphology with a magnet, it is important to eliminate the migratory ability of cells, leaving only the ability to deform mechanically due to the elongation of cells towards the magnetic floor. Because animal cells are highly plastic and are capable of actively migrating on plastic toward a magnetic field, the degree of their “stretching” may be minimal or absent.

The statistical analysis of cells shows that cells are oriented along magnetic field lines, and this effect is especially pronounced closer to the magnet. Thus, the cells have only two reactions left—accelerated migration and a change in morphology and orientation toward the magnetic field. The cellular response to magnetic nanoparticles depends on the type of cytoskeletal component and the biological response to each of these effects may be different. The tension effect of actin filaments, microtubules, and vimentin is different, which is determined by the unequal contributions to cell migration and orientation. Actin, as the main player in cell migration and shape maintenance, did not show significant changes in cell shape and orientation. The effect of magnetic nanoparticles on vimentin and tubulin has a reliable effect on the orientation of cells towards the magnet. Probably, the orientation of cells in a magnetic field is possible only with mechanical action on microtubules and intermediate filaments.

During experiments with 3D spheroids, the orientation of the spheroid “body” towards the magnet was noted. However, the morphology of the spread cells remained unchanged, likely due to their strong contact with the plastic culture surface. This suggests that cells with a smaller amount of magnetic nanoparticles can spread freely out over the entire surface of the Petri dish. Large cell aggregates can deform in a magnetic field and lengthen toward the magnet.

The result shows that cells loaded with magnetic nanoparticles can react to a magnetic field by exhibiting minor morphological changes, such as stretching toward the field. These changes are temporary and occur primarily at the initial stage of magnetic field exposure. The limited extent of these changes is likely due to the cells’ strong adhesion to the extracellular matrix and the plastic substrate. It appears that the mechanical forces generated by the magnetic nanoparticles are insufficient to cause significant or visible deformation under a constant magnetic field.

## 4. Materials and Methods

### 4.1. Nanoparticle Synthesis

Used magnetic nanoparticles of the composition of iron core–carbon shell (Fe@C) and Fe_3_O_4_ nanoparticles are modified with functional groups that provide hydrophilicity and cross-linking with proteins.

Iron–carbon magnetic nanoparticles were synthesized and modified in the Laboratory of Applied Magnetism of the M.N. Mikheev Institute of Metal Physics using the gas-phase technique [20]. The iron wire was evaporated in a flow of inert gas (argon) with the addition of hydrocarbons (butane, propane, isobutane) by a high-frequency alternating electromagnetic field. On molten iron nanodroplets, the catalytic decomposition of hydrocarbons occurred with the formation of a carbon shell, after which the nanoparticles were captured by a fabric filter. Surface modification was performed using aryl-diazonium derivatives [21,22] (diazonium derivative 4-aminophenylacetic acid, Sigma Aldrich, St. Louis, MO, USA), which were processed for 30 min using a submersible ultrasonic activator, subsequently separated by magnets, and then washed. The nanoparticle saturation magnetization was 90 emu/g, and the size according to TEM data was ~10 nm.

Fe_3_O_4_ MNPs were synthesized as specified in Refs. [23,24]. Briefly, a saturated aqueous solution of ammonia was added to a solution of FeSO_4_ × 7H_2_O and FeCl_3_ × 6H_2_O in H_2_O (distilled water) with stirring and sonication in an ultrasonic bath (40 °C). After 10 min, the particles were washed on a magnet with H_2_O to neutral pH and dispersed in H_2_O. To functionalize the surface, a solution of PMIDA in H_2_O was added to the MNPs. The colloidal solution was mixed using an overhead stirrer. MNPs were centrifuged at 25,000 rpm for 15 min, washed with H_2_O, and dispersed in 50 mL of H_2_O. The nanoparticle suspension was filtered through 0.22 μm PTFE syringe filters (Jet Biofill, Guangzhou, China) and diluted with culture medium.

### 4.2. Carbodiimide Cross-Linking Nanoparticles with Proteins

#### 4.2.1. Nanoparticle Modification

Carboxylated nanoparticles modified the surface of MNPs with antibodies using the carbodiimide conjugation method [25]. Primary antibodies to vimentin (Cell Marque, 347R-25), polyclonal antibodies to beta-actin (PA1-183, Thermo Fisher Scientific, Waltham, MA, USA, and antibodies to acetylated tubulin (T7451, Sigma-Aldrich, St. Louis, MO, USA) were used. The choice of acetylated antibodies to tubulin is determined by the fact that acetylated forms of tubulin are associated with stabilizing modifications of microtubules [26]. Ig G + H with a fluorophore AF-488 (A-11013, Thermo Fisher Scientific, Waltham, MA, USA) was used as secondary antibodies.

To activate carboxyl groups on nanoparticles, EDC (39391, Sigma Aldrich, St. Louis, MO, USA) and NHS (130672, Sigma Aldrich, St. Louis, MO, USA) were used in a mass ratio of 1:3 and a mass ratio of EDC to nanoparticles of 6:23. The activation reaction of nanoparticles pretreated with an ultrasonic dispersant was carried out at RT for 20 min in the MES buffer (pH 6,0). To remove reaction byproducts and free EDC and NHS molecules, the solution was centrifuged for 5 min at 21,000 rcf at room temperature (RT), and the supernatant was removed. Buffer and antibodies were mixed with the residue in ratios of 1:23–2:23 to the volume of nanoparticles and incubated for 30 min at RT. The nanoparticle carboxyl groups formed an amide bond with the amino group of the antibodies. To remove unbound components from the reaction solution, the solution was centrifuged for 5 min at 21,000 rcf, the supernatant was removed, and the modified nanoparticles were diluted with serum-free culture medium.

#### 4.2.2. Nanoparticle Modification with Protein G

Nanoparticle conjugation was performed with carbodiimide conjugation method [25].

Modification was performed in two stages. Firstly, nanoparticle surface carboxyl groups were activated with EDC and sulfo-NHS solutions in 0.1 M MES buffer solution (pH 5.0). A total of 1.8 mg of nanoparticles dissolved in 45 μL of distilled water 120 μL of EDC/sulfo-NHS in MES was added (3 mg of sulfo-NHS and 6 mg EDC). The nanoparticle solution was treated with an ultrasonic dispersant and carried out at RT for 15 min.

To remove unconjugated cross-linker reagents, nanoparticle solution was centrifuged for 5 min (15,000× *g*), supernatant was removed, and 450 μg of protein G dissolved in 600 μL of borate buffer (0.4 M H_3_BO_3_, 70 mM Na_2_B_4_O_7_·10H_2_O, pH 8.0) was added. Nanoparticles were incubated overnight at 4 °C, centrifuged due to removing unconjugated protein G, and dissolved in water.

#### 4.2.3. Nanoparticle Modification with Protein G and Cy5 Fluorophore

A total of 1 mg of nanoparticles, 1 mg of EDC, and 1 mg of NHS was added in 100 μL of 0.1 M MES; pH 6.0. The solution was immersed in an ultrasonic bath and incubated for 30 min at RT. An amount of 1 mg of protein G in PBS and 100 μL of borate buffer pH 8.0 were added and ultrasonicated for 30 min at RT. Next, Cy5 fluorophore 20 μg was added and incubated for 8 h overnight. To remove contaminants from the solution, the mixture was centrifuged for 10 min at 21,000 rcf at room temperature, after which the supernatant was removed. This process was then repeated twice.

#### 4.2.4. Nanoparticle–Protein G–Antibody Conjugation

The nanoparticle solution was sonicated with an ultrasonic bath and mixed with antibodies (or a 1% BSA solution) and PBS (10 μL of protein G-modified nanoparticle solution, 4 μL of antibodies, and 190 μL of sterile PBS). The mixture was incubated for 15–30 min at RT, centrifuged for 5 min (15,000 rpm) to remove unlinked proteins, and dissolved in 200 μL of PBS. Before being added to a cell dish, the resulting solution was diluted to 1 mL with a cultural medium without FBS. The microtube with thenanoparticle solution was sonicated before being added to cells to remove any possible nanoparticle aggregates.

### 4.3. Paper Chromatography of Antibody-Bound Nanoparticles

Nanoparticles were used in two concentrations: 0.25 mg/mL and 0.625 mg/mL (2.5 μL and 6.25 μL of 5 mg/mL initial solution, respectively). Antibodies to BSA (A11133, Thermo Fisher Scientific, Waltham, MA, USA) were added in 50 μL of PBS in three concentrations: 0.04, 0.16, and 0.4 mg/mL (1, 4, and 10 mL of initial 2 mg/mL solution, respectively) for different cross-linking times (30, 60 min). An amount of 2.5 µL of 10% BSA solution (A8022, Sigma-Aldrich, St. Louis, MO, USA) in PBS pH 7.4 (P4474, Sigma-Aldrich, St. Louis, MO, USA) was added to a nitrocellulose membrane and an absorbent pad (UniSart CN 140, Sartorius AG, Göttingen, Germany) [27]. Then, the mobile phase was prepared. It contained 2 μg or 5 μg of Fe nanoparticles crosslinked with antibodies and 0.8 μg of casein (4 μL of 0.2 mg/mL casein in PBS solution, made by adding casein powder (C3400, Sigma-Aldrich, St. Louis, MO, USA) in PBS, mixing on magnetic stirrer with heating for 4–5 h, and filtering through paper filter). The total volume of the mobile phase was brought up to 20 μL with PBS. The membrane was lowered into the mobile phase, which moved up along it within 30 s and reached the line, where BSA antibodies reacted with the BSA. Due to the dark color of the nanoparticles, it is possible to visually detect bands on a chromatographic strip. Thus, the thickness of the band will correlate with the number of reacted MNP–antibody complexes with the antigen and, accordingly, the preservation of the affinity of the antibodies.

### 4.4. Cytoskeleton Labeling

The cytoskeleton was labeled by staining with antibodies, a complex of antibodies and nanoparticles, or with phalloidin.

Regarding immunocytochemistry, cells were fixed with ice-cold methanol for 10 min, permeabilized with 0.5% Triton X-100 in PBS for 15 min, and incubated overnight at 4 °C with primary antibodies in PBS. The following antibodies were used: β-actin antibody (dilution 1:200; PA1-183, Thermo Fisher Scientific, Waltham, MA, USA), vimentin antibody (dilution 1:200; A19607, ABClonal, Woburn, MA, USA), and α-tubulin acetyl K40 (dilution 1:200; ab179484, Abcam, Camridge, UK). The cells were washed three times with PBS and incubated with fluorophore-conjugated secondary antibody solution (dilution 1:2000; A-11013, Thermo Fisher Scientific, Waltham, MA, USA) in the dark for 1 h at room temperature. Then, cells were washed three times with PBS, incubated with DAPI (D1306, Thermo Fisher Scientific, Waltham, MA, USA) in the dark for 5 min, washed three times with PBS, and stored in PBS with 0.1% sodium azide.

For phalloidin staining, cells were fixed with 4% PFA for 15 min at room temperature and permeabilized in the same way as in the case of immunocytochemistry. Samples then were incubated with AF-488 phalloidin (A12379, Thermo Fisher Scientific, Waltham, MA, USA) for 15 min at room temperature in the dark.

Labeling with nanoparticles was fulfilled with two protocols: (1) pure antibodies were added to cells followed by nanoparticles, bound only with protein G and AF-430 dye; or, (2) nanoparticles, pre-bound to protein G and AF-430 dye, were cross-linked with antibodies, and thus the so-called complexes were created, which was then followed by cell labeling with these complexes. An amount of 50 μg nanoparticles was cross-linked with 1 μg AF-430 (10820, Lumiprobe, Moscow, Russia) and 50 μg protein G, and then conjugated with 0.2 μg beta-actin antibodies for 30 min at RT. Cells were incubated with antibodies (1 and 2 protocols) for 1 h at 37 °C and then washed three times with PBS for 2 min. MNP–protein G–AF-430 (protocol 2) complexes were added to the antibody-containing wells, incubated for 15 min at RT, and washed three times with PBS. After that, cells were labeled with antibodies to beta-actin, vimentin, and tubulin in confocal dishes for 1 h at 37 °C.

### 4.5. Magnetic Systems

The article used magnetic systems with a vertical gradient to accelerate the uptake of nanoparticles by cells and bypass lysosomal uptake and magnetic systems with a lateral gradient, causing a mechanical force to cells with magnetic nanoparticles.

**Figure 10:**
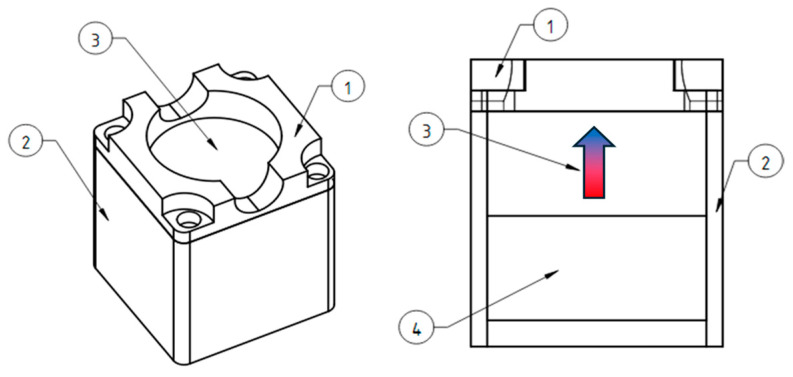
Diagram of the design of magnetic systems with a vertical gradient; the arrow indicates the direction of magnetization of the magnets. (**1**) plastic cover that allows centering the cup on the magnet, (**2**) plastic housing of the magnet system, (**3**) permanent magnet, (**4**) magnetically soft cylinder allowing to smooth the shape of the magnetic field.

The vertical gradient magnetic system was made of cylindrical permanent NdFeB magnets with a diameter of 50 mm and a height of 20 mm (Figure 10). There is a plastic protective cover (1) on top that allows you to center the Petri dish on the magnet, which is bolted to the plastic body (2). Inside, there is a cylindrical magnet (3) and a magnetic field concentrator of similar size (4) made of a soft magnetic alloy, which make the field more uniform along the edges of the magnetic system.

**Figure 11:**
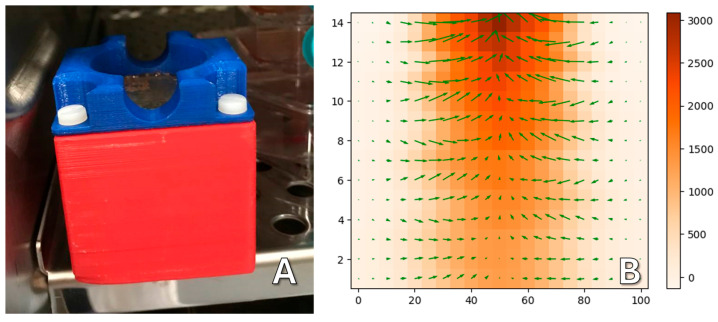
(**A**) Photograph of the assembled magnetic system in a cell incubator and measured topography of the magnetic field in the work area (values are given in Oe); (**B**) green arrows indicate the direction of the field gradient.

The 3D-printed plastic case is necessary for the safety of users (Figure 11A). Magnets of this size are attracted to each other and to ferromagnetic materials with great force and can seriously injure a person if used carelessly. The measured topology of the magnetic field is shown in Figure 11B; the vertical field gradient is about 1 × 10^4^ T/m.

**Figure 12:**
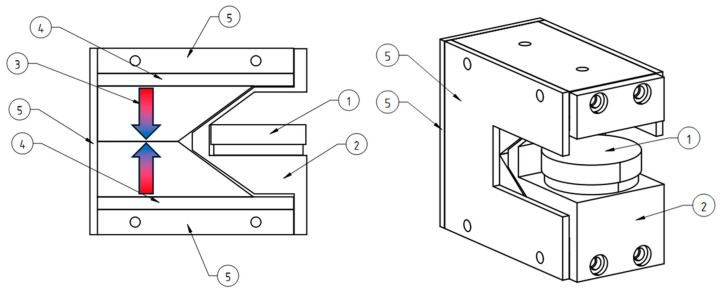
Diagram of the design of magnetic systems with a lateral gradient; the arrow indicates the direction of the magnetization of the magnets. (**1**) 35 mm culture dish with biological sample, (**2**) plastic support for the dish, centering it in the magnetic field, (**3**) permanent magnets, (**4**) magnetic field concentrators made of magnetically soft material, (**5**) aluminum plates holding the structure.

Lateral gradient magnetic systems were made from beveled permanent NdFeB magnets. This shape forms a magnetic field with a uniform gradient to achieve uniform forces to cells in a culture. A sketch of the magnetic system is shown in Figure 12. A Petri dish (1) with cells is placed on a plastic holder (2) located between two magnets (3). Above and below the magnets, there are magnetic field concentrators made of a soft magnetic iron alloy (4). The oppositely directed magnetic field leads to the appearance of a repulsive force between the magnets, so that the magnetic system maintains its integrity; it is held externally by an aluminum housing (5).

**Figure 13:**
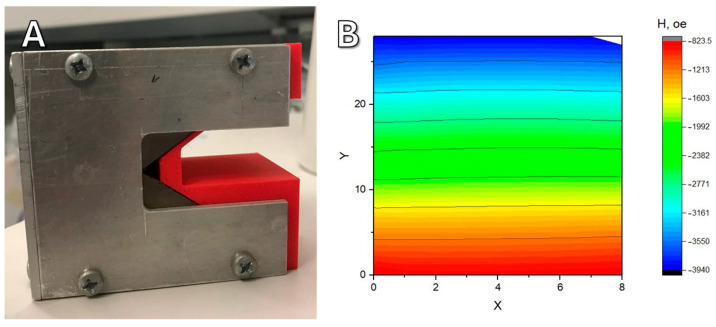
(**A**) Photograph of the assembled magnetic system. (**B**) Measured topography of the magnetic field in the work area.

The magnetic system is held together by screws screwed into the metal parts of the structure (Figure 13A). Using a Hall sensor, the topography of the magnetic field was taken in the working area of the magnetic system at the height where the dish with cells was located (Figure 13B). The measured gradient was 20 T/m. At the size of an individual nanoparticle of about 10 nm, this gives about 0.5 piconewtons of force pulling toward the magnet. According to some data [28,29], this is enough to have an effect on individual cell components. However, it should be noted that under real physiological conditions, particles can cluster [30], which may cause the force to increase significantly. In addition, several particles can bind to one or another compartment, especially such extended ones as the cytoskeleton.

### 4.6. Cell Culture

The hTert MSC ASC52telo cell line and human bone marrow mesenchymal stem cells (174H) obtained by biopsy from an adult male were used in the experiments. MSC lines are not characterized by cell polarization in space, which is very important when choosing a model cell line. This cell line is ideal for determining if cells can orient themselves in space. This research was conducted in accordance with the Declaration of Helsinki following the permission of the local ethics committee. The cells were cultured on a commercially available medium DMEM (C410п, Paneco, Moscow, Russia) with 10% FBS (Biosera, Cholet, France), L-glutamine, 100 U/mL penicillin, and 100 μg/mL streptomycin (Thermo Fisher Scientific, Waltham, MA, USA). The cells were cultured in T25 flasks (Jet Biofil, Guangzhou, China) or 35 mm confocal Petri dishes (Jet Biofil, Guangzhou, China) in an incubator at a temperature of 37 °C in a humidified atmosphere with 5% CO_2_. A 0.25% trypsin-EDTA solution (P036p, Paneco, Moscow, Russia) and a Versene solution (R080p, Paneco, Moscow, Russia) were used for cell reseeding. The working confluence for cell experiments ranged from 70% to 100%.

### 4.7. Cell Incubation with Nanoparticles

Human bone marrow mesenchymal stem cells (174H) and hTert MSC ASC52telo cells were used. Dansylcadaverine, which suppresses vesicular transport in cells [31], was used to inhibit the endocytosis of nanoparticles. The cells were incubated with 1000 μL of 100 mM monodansylcadaverine, either with or without 1.5 μL of integrin α3 antibodies (SC-374242, Santa Cruz Biotechnology, Dallas, TX, USA) for 60 min. Then, the cells were incubated with a nanoparticle solution on magnetic systems with a vertical gradient for 60 min, followed by incubation with nanoparticle solution for 30 min without magnets. After incubation, nanoparticle solution was removed and the cells were washed with PBS one to two times to eliminate unabsorbed nanoparticles. Depending on the experimental protocol, the cells were detached with trypsin-EDTA and reseeded in a new cultural dish or left in a previous dish.

The edge of the dish closest to the magnetic system was marked. The cells were incubated on magnetic systems with a lateral gradient for 20 h.

### 4.8. Spheroid Production with Magnetic Nanoparticles

To form the spheroids, hTert MSC ASC52telo cells and human bone marrow mesenchymal stem cells (174H) were used. The cells were first incubated with 1000 μL of 100 mM monodansylcadaverine for 60 min. Then, they were incubated with nanoparticle solution on a magnetic system with vertical gradient for 60 min, washed with PBS to remove any remaining on the cell surface nanoparticles, detached with trypsin-EDTA, and centrifuged to remove trypsin solution. The cells were resuspended with 200 μL of cultural medium and placed in agarose molds. Approximately 2 × 10^6^ cells were used for each mold. The agarose molds for spheroids were made of 2% PBS agarose gel using silicone molds. The molds were then incubated in culture medium for at least 30 min or longer [32]. The cells were incubated in the molds for a day before spheroid transfer. The spheroids were transferred from the mold to the Petri dish containing a complete nutrient medium for 30 min to allow the spheroids to attach to the surface of the dish. Then, they were placed in magnetic systems with a lateral gradient overnight.

### 4.9. Measurement of Nanoparticle Uptake in Cells by NMR Relaxometry

To prevent endosome formation, the cells were incubated with 100 mM monodansylcadaverine solution for 60 min before incubation with nanoparticles, and then the nanoparticle solution was added to the cells. The process of the uptake of nanoparticles by the cells was studied using NMR relaxometry, as described in Refs. [33,34]. The cells were seeded at 70% confluency into a culture vessel and incubated for 24 h in culture medium with nanoparticles. The relaxation time of samples (100 μL each) was measured using an NMR relaxometer. To obtain reproducible results, the liquid was resuspended each time using a dispenser. The value of 1/T2 is linearly proportional to the concentration of magnetic nanoparticles in the liquid and is presented in the graphs [22].

### 4.10. Lysis Protocol

The cells were detached from the plastic surface using trypsin and then centrifuged and placed in an ultrasonic bath for 30 s (FB11203, Thermo Fisher Scientific, Waltham, MA, USA) to partially disrupt the cell membranes and make the cytoskeletal fibrils more accessible. Antibodies to vimentin, actin, and tubulin cross-linked with iron oxide nanoparticles (5 μL of MNP solution (2,3 mg/mL) was mixed with 2 μL of antibody stock solution in 50 μL PBS) were added to the cell lysate, incubated in a thermostat at 37 °C for 30 min, and labeled by secondary antibodies with a fluorophore AF-488, and were then added. The resulting suspension was carefully washed using magnetic separation for 1–2 min, and then the remaining liquid was carefully removed with a dispenser and replaced with 100 μL of PBS and placed in a magnetic system with a lateral magnetic field.

### 4.11. Cell Migration

To study migration, cells were cultured in dishes with agarose molds. Molds were made according to the method described in Ref. [35]. Master-forms were printed using MSLA 3D printer Photon Mono (Anycubic, Shenzhen, China) and filled with two-component cast silicone. After the silicone solidified, the forms were washed with isopropyl alcohol and stored in zip-lock bags.

**Figure 14:**
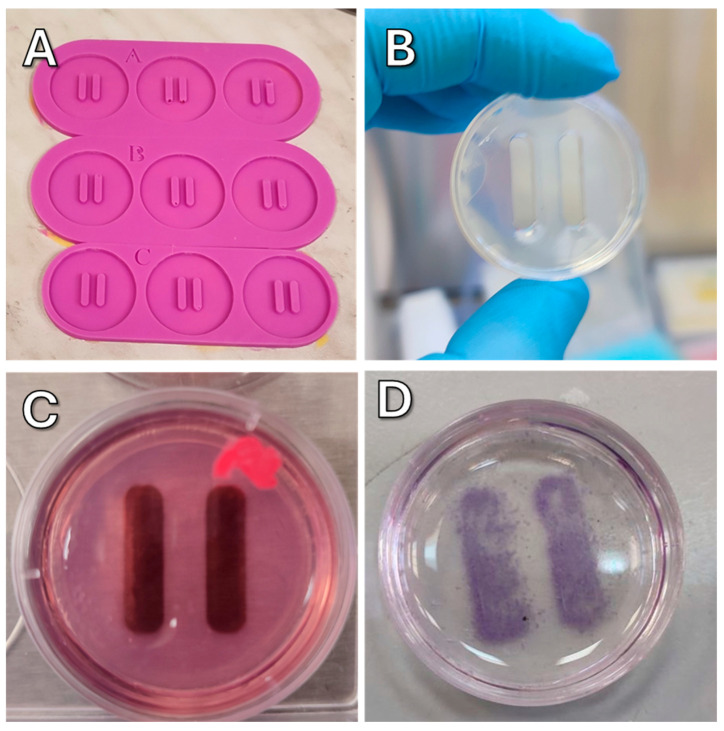
(**A**) Silicone molds. (**B**) Agarose mold mounted in a Petri dish. (**C**) Suspension of nanoparticle-loaded cells poured into the cells of agarose mold. (**D**) Cells after mold removal (H&E staining).

To make agarose molds, a molten solution of 2% agarose in PBS was poured into the silicone mold (Figure 14A). After solidification, which lasted for 5–10 min depending on ambient temperature, the agarose mold (Figure 14B) was transferred to a dish. Before use, the agarose mold was soaked in complete culture medium for 30 min (Figure 14C).

Cells were seeded into 35 mm cell culture dishes with molds and incubated overnight, and then molds were carefully removed from dishes. The cells were then incubated with magnetic nanoparticles, the same as described above. The Petri dishes were placed in a magnetic system with a lateral magnetic field and incubated. Control samples were incubated without a magnetic field.

### 4.12. Migration and Polarity Experiments in Lateral Gradient Magnetic Systems

A solution of modified nanoparticles was added to the serum-free culture medium in a Petri dish containing cells. The cells were incubated with the nanoparticles for 1 h, and then the cell layer was gently washed with a complete culture medium and the medium was replaced with a complete medium with 10% FBS. To suppress vesicular transport, cells were treated with the endocytosis inhibitor dansylcadaverine (D4008, Sigma-Aldrich, St. Louis, MO, USA), and to suppress cell migration, cells were treated with Integrin beta-1 antibodies (MA5-27900, Thermo Fisher Scientific, Waltham, MA, USA). After 30 min in the CO_2_ incubator, the Petri dish was placed on a lateral gradient magnetic system with a mark indicating the orientation of the dish. Dishes with cells were incubated for 20 h on a lateral magnetic system.

### 4.13. Image Acquisition and Processing

Confocal imaging was performed using a Nikon eclipse Ti_2_ microscope. Images were obtained with 10× objective. All recordings were performed using a 131.5 μm pinhole diameter. The following laser lines were used for fluorescence excitation: a 405 Diode (405 nm) and an argon laser (488 nm).

**Figure 15:**
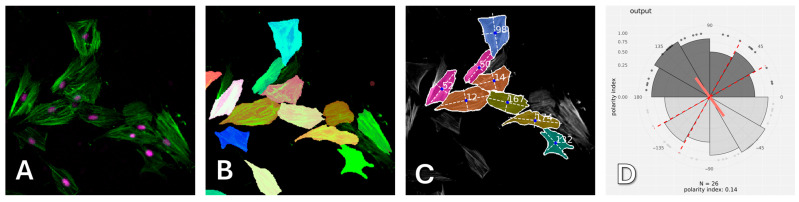
Processing images to estimate their orientation and polarization. (**A**) Original image in two channels (green—cytoskeleton, magenta—nucleus); (**B**) superimposed masks obtained in CellPose; (**C**) orientation visualized with PolarityJam; (**D**) cell polarization graph plotted with PolarityJam.

Cell orientation was assessed using PolarityJAM [18]. To use PolarityJAM, cells were first segmented using CellPose 3 [36]. After that, the resulting masks and raw images were processed using PolarityJAM. Plots showing cell orientation were plotted using an online tool from the PolarityJam suite, version 1.23.5, (Figure 15).

## Data Availability

Data is contained within the article and Supplementary Materials.

## Supplementary Materials

The following supporting information can be downloaded at: https://www.mdpi.com/article/10.3390/ijms26115330/s1.
