# Supplementary material for: Effect of a Constant Magnetic Field on Cell Morphology and Migration Mediated by Cytoskeleton-Bound Magnetic Nanoparticles
Source: Int J Mol Sci. 2025 Jun 1;26(11):5330. doi: 10.3390/ijms26115330 (PMC12155149; doi:10.3390/ijms26115330)
Supplement: Supplementary file 1 [file ijms-26-05330-s001.zip › ijms-3602207-supplementary2.pdf]

Supplementary

Supplementary Figures

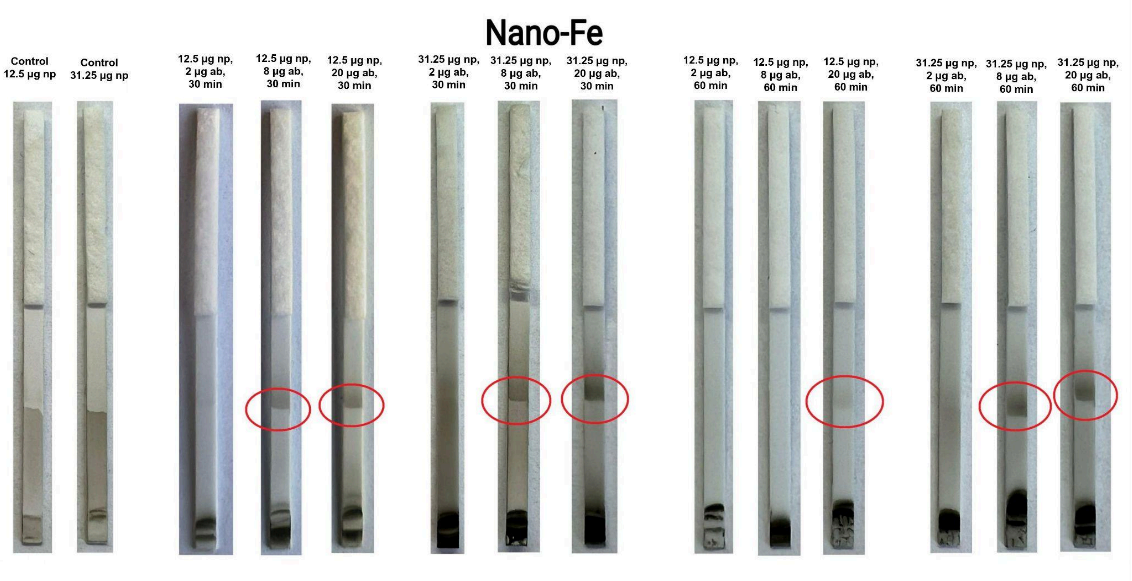

Figure S1. Optimization of cross-linking conditions for Fe NPs (5 mg/ml). The binding site of antibodies cross-linked to BSA nanoparticles is circled in red.

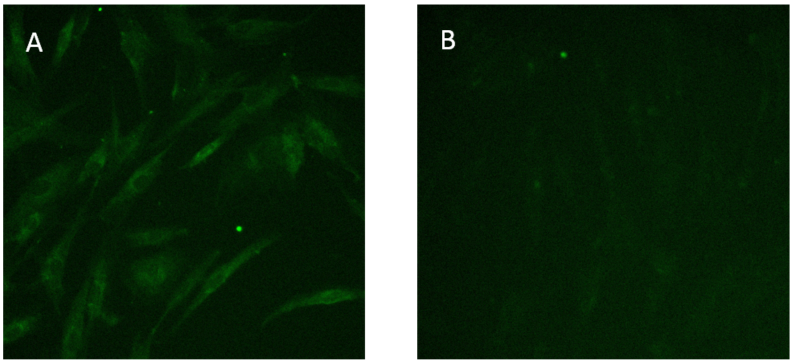

Figure S2. Immunocytolabeling with antibodies to actin. A: labeling with complexes of Fe nanoparticles/protein L/AF-430/antibodies; B: Sequential labeling with primary antibodies and complexes of Fe nanoparticles/protein L/AF-430.

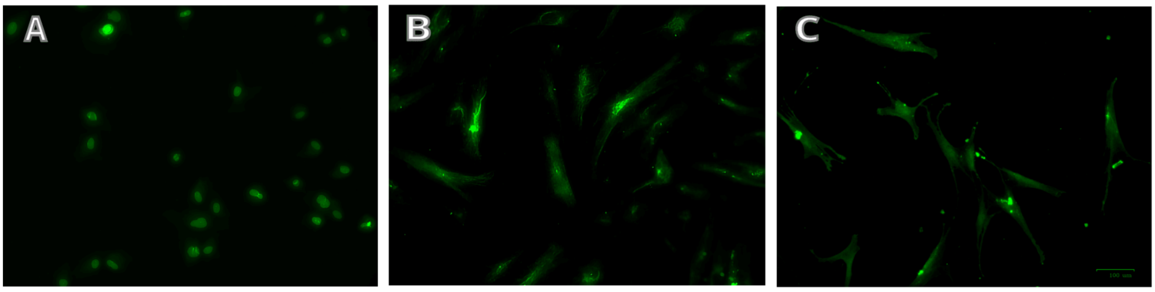

Figure S3. immunohistochemistry. 174H cells. A: antibodies to vimentin; B: antibodies to alpha-acetyl-tubulin, C: antibodies to beta-actin. Cells were fixated with ice-cold methanol.

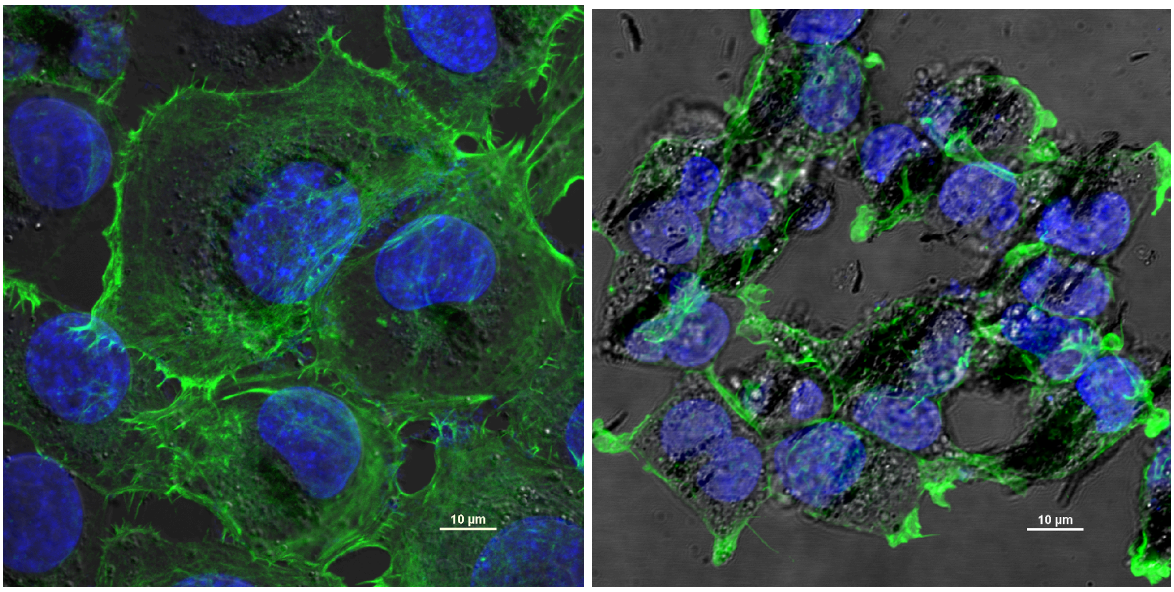

Figure S4. MSC with magnetic nanoparticles without magnetic field and in magnetic field

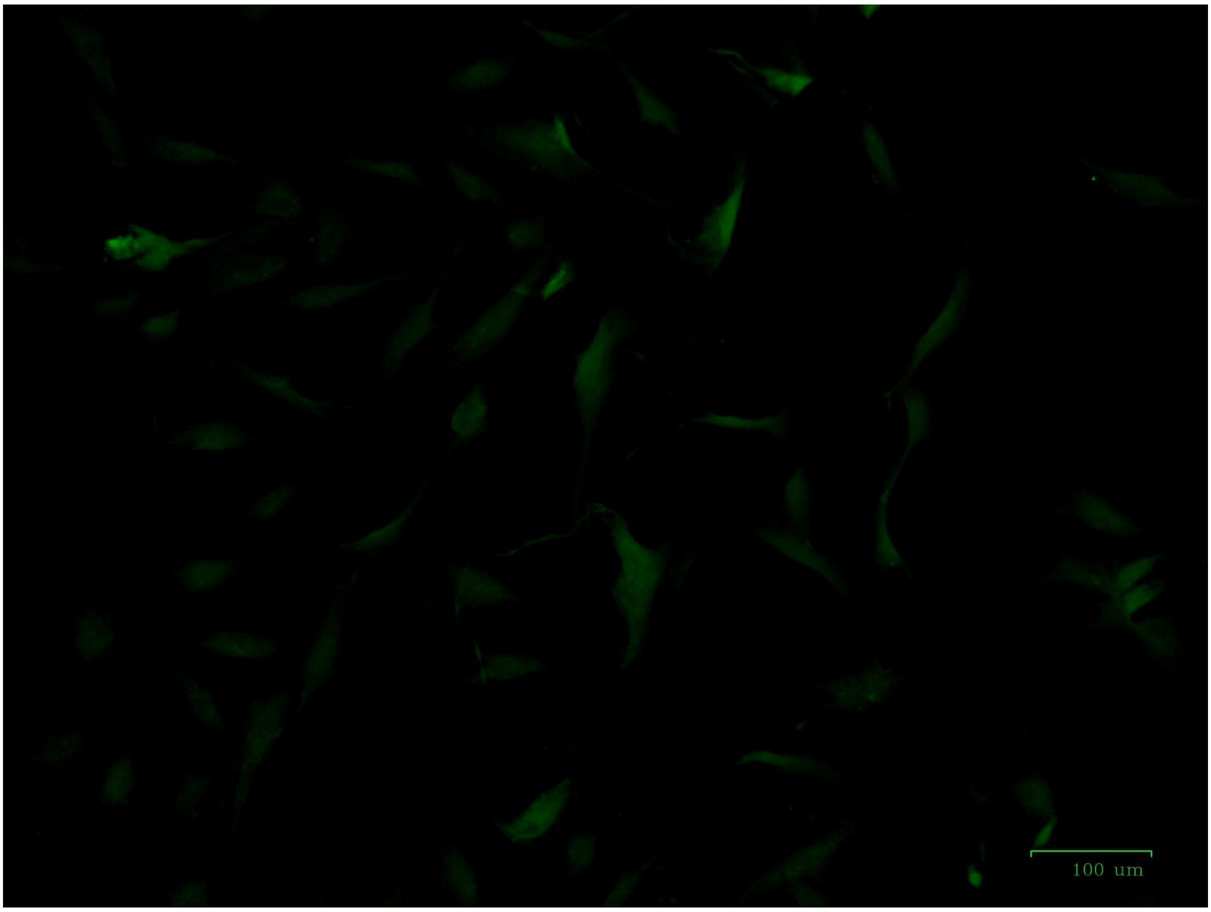

Figure S5. Immunocytochemistry performed with alpha 3-integrin antibody.
